# Impacts from Economic Development and Environmental Factors on Life Expectancy: A Comparative Study Based on Data from Both Developed and Developing Countries from 2004 to 2016

**DOI:** 10.3390/ijerph18168559

**Published:** 2021-08-13

**Authors:** Zhiheng Chen, Yuting Ma, Junyi Hua, Yuanhong Wang, Hongpeng Guo

**Affiliations:** 1 College of Northeast Asian Studies, Jilin University, No. 2699 Qianjin Street, Changchun 130012, China; czhjlu@163.com; 2 College of Biological and Agricultural Engineering, Jilin University, No. 5988 Renmin Street, Changchun 130022, China; mayuting66666@163.com (Y.M.); huajy1819@mails.jlu.edu.cn (J.H.); wangyh1819@mails.jlu.edu.cn (Y.W.)

**Keywords:** life expectancy, multiple regression models, environmental factors, socio-economic development

## Abstract

Both economic development level and environmental factors have significant impacts on life expectancy at birth (LE). This paper takes LE as the research object and selects nine economic and environmental indicators with various impacts on LE. Based on a dataset of economic and environmental indicators of 20 countries from 2004 to 2016, our research uses the Pearson Correlation Coefficient to evaluate the correlation coefficients between the indicators, and we use multiple regression models to measure the impact of each indicator on LE. Based on the results from models and calculations, this study conducts a comparative analysis of the influencing mechanisms of different indicators on LE in both developed and developing countries, with conclusions as follow: (1) GDP per capita and the percentage of forest area to land area have a positive impact on LE in developed countries; however, they have a negative impact on LE in developing countries. Total public expenditure on education as a percentage of GDP and fertilizer consumption have a negative impact on LE in developed countries; however, they have a positive impact on LE in developing countries. Gini coefficient and average annual exposure to PM_2.5_ have no significant effect on LE in developed countries; however, they have a negative impact on LE in developing countries. Current healthcare expenditures per capita have a negative impact on LE in developed countries, and there is no significant impact on LE in developing countries. (2) The urbanization rate has a significant positive impact on LE in both developed countries and developing countries. Carbon dioxide emissions have a negative impact on LE in both developed and developing countries. (3) In developed countries, GDP per capita has the greatest positive impact on LE, while fertilizer consumption has the greatest negative impact on LE. In developing countries, the urbanization rate has the greatest positive impact on LE, while the Gini coefficient has the greatest negative impact on LE. To improve and prolong LE, it is suggested that countries should prioritize increasing GDP per capita and urbanization level. At the same time, countries should also work on reducing the Gini coefficient and formulating appropriate healthcare and education policies. On the other hand, countries should balance between economic development and environmental protection, putting the emphasis more on environmental protection, reducing environmental pollution, and improving the environment’s ability of self-purification.

## 1. Introduction

Health is considered as “one of the fundamental rights of human beings” by the World Health Organization (WHO). Life expectancy is not only the main rating index of human beings’ health, it is also a comprehensive index to evaluate the level of economic development, education, healthcare systems [1], and environmental quality. As one of the crucial health indicators of the World Health Organization (WHO), life expectancy is considered as the most important indicator that reflects human beings’ livelihood [2,3,4,5].

In most parts of the world, life expectancy has increased significantly. Global life expectancy has increased from 67.2 years in 2005 to 70.8 years in 2015 (Source: United Nations Statistical Yearbook, 2017 edition. Data refers to a five-year period preceding the reference year). Improving life expectancy as well as people’s health and wellbeing has become an essential goal for both the United Nations and various national governments [6,7]. The United Nations has been actively involved in the promotion of human beings’ health, providing healthcare services, improving the urban environment, and providing assistance in developing countries (United Nations Economic and Development Website: https://www.un.org/chinese/esa/health.htm) (accessed on 9 February 2021).

Since the beginning of the 21st century, the world’s economy has reached an unprecedented level of development, bringing benefits to the wellbeing and development of mankind. However, with the rapid economic development, climate change and environmental pollution also now pose a huge threat to the health of mankind. Hence, it has become a huge challenge for mankind to strike a balance between achieving rapid economic growth and protecting the environment to improve the quality of life.

At present, the development of the world’s economy has slowed down, [8] with the developed countries leading the economic growth. Meanwhile, developing countries, especially those in Asia, have become fast growing areas. In addition, when it comes to coping with environmental and climatic changes, developed countries and developing countries have different strategies and technological readiness. Therefore, there are many differences between developed countries and developing countries in terms of economic development level and environment factors. This paper hypothesizes that economic development level and environmental factors have different influencing mechanisms on life expectancy in both developed countries and developing countries. This study will demonstrate and analyze this hypothesis.

We have found through our extensive research that most of the existing studies contain qualitative analyses of a single factor or a few factors. There is a lack of quantitative analyses of multiple factors, leading to the situation that the predominant influencing factor of life expectancy cannot be accurately identified [5,9]. In addition, existing studies tend to ignore the differences in the level of economic development and the role of environmental factors among different regions. Besides, there are differences in the impacts from changes in external factors on the life expectancy of populations in different geographical areas. Hence, this paper argues that it is necessary to study the differences in the impacts from the level of economic development and environmental factors on life expectancy in both developed countries and developing countries.

By studying the differences in life expectancy between developed countries and developing countries, it will help the United Nations to improve its ongoing work on the promotion of human health and the assistance of mankind. It will also be helpful for various national governments to formulate more effective strategies to prolong life expectancy, to improve the quality of life, and to strike a balance between economic development and environmental protection. At the same time, all countries should join together to actively promote sustainable economic development, to jointly tackle environmental pollution and global climate change, to create a community of a shared future for mankind, and to ultimately achieve the health, longevity, and sustainable development of mankind.

## 2. Literature Review

### 2.1. Study on the Factors That Affect Life Expectancy

Previously, researchers believed that the relevant factors affecting life expectancy were mainly environment factors, social factors, economic factors, and lifestyle factors. These factors were spatially varying, and they included economic development, medical conditions, population structure, and geographical locations [6,10]. These factors had an indirect impact on life expectancy by affecting a person’s wellbeing. Studies on the impacts on life expectancy from economic development level and environmental factors were relatively abundant. The researches believed that economic development and environmental factors could have impacts on life expectancy [6,11].

Existing research works have been open to controversy concerning the key determining factor of life expectancy. For example, Zha [9] analyzed the life expectancy in Tibet in China, and he believed that the major factors determining the life expectancy were socioeconomic factors and geological environment factors. Ronald [12] believed that the social economy played an important role in determining life expectancy in the early stages of development. However, it was replaced by diet and living habits after the economic development reached a certain level. Cockerham [13] argued that the decisive factor of changing life expectancy in eastern Europe was lifestyle;

In conclusion, there have been abundant studies on factors affecting life expectancy; however, few studies have compared economic development with environmental factors to analyze the intensity of their effects on life expectancy.

### 2.2. Research on the Impact from Economic Development Level on Life Expectancy

Economic development level has an important effect on life expectancy. Studies have shown that people with high socioeconomic status and those from well-established families tend to have a higher life expectancy [14,15].

Research works on the impacts from economic development on life expectancy have focused on healthcare expenditures and income per capita. The tremendous improvement in longevity in the 20th century was largely due to increased spending on healthcare and better economic conditions [16]. Governments could significantly increase the life expectancy of the population in their countries by increasing their expenditures on healthcare system. [6] In terms of income per capita, Chetty [17] believed that longer life expectancy in the US was largely associated with increased income level. However, the link between life expectancy and income varies greatly in different regions. Some scholars believed that there was a negative correlation between income inequality and life expectancy in both developing countries and developed countries, which led to a negative correlation between human capital inequality and human capital accumulation. [15,18] Wilson argued that income inequality was a major determinant of changes in life expectancy [19]. Reducing the socioeconomic disparities caused by external factors contributes to achieving equalization of life expectancy among those with corresponding incomes [20].

Some scholars believed that other variables of economic development such as GDP per capita [21,22], urbanization rate [21], and education level [20] can affect life expectancy to different extents. Huang [6] believed that there was a positive correlation between GDP per capita and life expectancy per capita. Kim [23] believed that there was a positive correlation between urbanization level and life expectancy. Michael [24] suggested that the life expectancy of an indigenous population could be extended by 12 years if the general education level of the population was improved. Haebong [25] argued that inequality in life expectancy, as measured by the Gini coefficient, has fallen sharply in South Korea over the past 20 years. In addition, some studies have shown that the pursuit of economic development in developing countries can have an adverse impact on the environment, such as industrial pollution, indirectly affecting the life expectancy of local residents [6,26,27]. However, environmental expenditure resulting from the accumulation of economic capacity can increase life expectancy [28].

The research works on the economic factors affecting life expectancy have provided the economic development direction for many countries from a theoretical level, with certain practical significance.

### 2.3. Study on the Impact from Environmental Factors on Life Expectancy

Environmental factors are important determining factors for the population’s wellbeing [11]. Most environmental factors, such as ecological resilience and environmental sustainability, are positively correlated with life expectancy, while a few factors, including biodiversity, are negatively correlated with life expectancy [11,29]. Current environmental conditions influence the life expectancy of the population at birth, while the cumulative changing circumstances continue to influence the remaining life expectancy of the population at different ages along the way [30].

Research on the impact of environmental factors on life expectancy has focused on air pollution. Short-term and long-term exposure to pollutants have a significant impact on premature death and reduced life expectancy [31]. Among them, particulate pollutants (PMs) have a particularly significant impact [32]. Anderson [33] believed that people living in an environment with high levels of particulate pollutants for a long time have a higher cardiovascular morbidity, and there was a certain degree of dose dependence.

Other researchers studied the impacts from different environmental variables on life expectancy, and Wuffle [34] compared the average temperature of all the states in the United States. The results showed that the lower the average temperature in November, the higher the life expectancy of the population in those states. Huang [6] conducted a comparison of different regions in China, and the results showed that the higher the annual average temperature, the longer the life expectancy of the local people. Laura [10] believed that the differences in natural geographical factors, such as climate, meant that the life expectancy of people in different regions varied greatly. Brunner [35] suggested that there was a relationship between carbon dioxide emissions and life expectancy in countries with low-income to mid-income levels; however, the same trend did not show up in high-income countries. Scholars, represented by Mariani [29], have noticed the existence of the “environmental poverty trap”; that is, the trap of “low life expectancy and low environmental quality”. He believed that it would occur in developing countries more often. Wu [26] argued that the “environmental poverty trap” might happen to both developing countries and developed countries due to their proximity to each other, where environmental conditions interact and are shared.

The analysis of the impact from environmental factors is not only a theoretical breakthrough, it also carries practical significance to improve human beings’ awareness of environmental protection. For certain areas, a breakthrough in environmental conditions must be made through improvement of the public environment to achieve a positive effect from environment to life expectancy [36]. At present, there are few studies on the effects of chemical fertilizer consumption and forest areas on life expectancy in different regions. This paper will make a comparative analysis of the effects from these two factors on life expectancy.

### 2.4. Research on Regional Differences in Life Expectancy

Some studies have put forward calculation methods of life expectancy, such as the weighted mean method of three dimensions and the Grevillie method. At present, life expectancy has been mainly calculated by constructing a life table [22,37].

The differences in life expectancy and local people’s behaviors are related to regional characteristics [38]. Changes in external factors such as healthcare expenditure and income inequality have different impacts on different age groups, genders, and national populations. Therefore, an increase in life expectancy does not mean that all groups will benefit from it [22,37].

There are abundant studies focusing on the influencing factors of life expectancy in developed countries. Cervantes believed that the most important factor affecting life expectancy in European countries was social security spending, followed by healthcare expenditures, GNI per capita, education levels, and environmental expenditures. [38] Spencer argued that there was a statistically significant correlation between infant mortality, income inequality, and social policy indicators in developed countries; however, further research was needed to substantiate this finding [39]. Torre argued that reducing income inequality through policies could improve health conditions [40]. Dierk argued that income inequality in developed countries added slightly to life expectancy [41].

At present, more and more studies have focused more on developing countries. David believed that, for developing countries such as Brazil, reducing income inequality was an important part of improving the health conditions and life expectancy of their population. [42] Lin believed that, with the passage of time, the influence of political systems on life expectancy will be less in developed countries, while other socioeconomic factors will have the opposite effects. [43] Peter believed that the factors traditionally considered to have a significant impact on life expectancy in developing countries were not significant in Nigeria. The government can improve life expectancy by improving the quality of healthcare expenditures and reducing the unemployment rate [44]. Sophie believed that urbanization had a negligible impact on life expectancy in developing countries, and governments should pursue appropriate healthcare policies [45].

Although there is plenty of existing research, few studies have compared factors affecting life expectancy between developing countries and developed countries.

### 2.5. Methods Review

In the existing studies, the main methods used to investigate the impacts on life expectancy were spatial Durbin models [46], standard error regression estimation models [21], and least squares regression models [6].

In addition to the three methods above, Paramita used a great likelihood estimation model to examine the determinants of life expectancy in Indonesia [47]. Bushnik constructed a bootstrap model to explain the persistence of the life expectancy gap in Canada [48]. Laura used the categorical model to analyze the idea that the geographical variations in life expectancy in the UK were mainly caused by poverty [49]. Zha quantitatively assessed the environmental influences on life expectancy in Tibet, China, using the Geodetector [9]. Kim used correlation coefficients and multiple regression models to assess the association and correlation between socio-ecological indicators and LE to investigate the impacts of socio-ecology on inequality in LE [50]. Multiple regression models have been widely used in LE research, providing more in-depth support for future studies.

### 2.6. Summary

More and more attention has been paid to the study of life expectancy. Existing studies have mainly focused on the work mechanisms and intensity of impact from a single influencing factor. Few studies have compared the impacts from economic development level and environmental factors. At the same time, the existing controversy regarding the significant determinants of life expectancy varies greatly in different literatures.

As for the regional differences in life expectancy, most existing studies have focused on a single country, especially those in developed regions, without comparing the developing countries with the developed countries. Due to the great difference between developed countries and developing countries in economic level, the response from developing countries to environmental changes was more obvious. Thus, it is necessary to build up a comparison between developed countries and developing countries.

Based on the analysis above, this paper compares the influence of economic development level and environmental factors on life expectancy in 10 developing countries and 10 developed countries. The results from this paper are of practical significance.

## 3. Hypothesizes

In both developed and developing countries, we hypothesize that significant positive correlations exist between *y*_1_/*y*_2_, LE per capita, and *x*_1_, GDP per capita; *x*_2_, urbanization rate; *x*_3_, current healthcare expenditures per capita (in USD); *x*_4_, total public expenditures on education (total public expenditures on education as a percentage of GDP); and *x*_9_, forest area (forest area as a percentage of land area). At the same time, we hypothesis significant negative correlations exist between LE per capita and *x*_5_, Gini coefficient; *x*_6_, average annual exposure to PM_2.5_ (micrograms per cubic meter); *x*_7_, CO_2_ emissions (metric ton per capita); and *x*_8_, fertilizer consumption (kilograms per hectare of arable land).

In order to compare the differences in developing countries and developed countries, we have established multiple linear regression models for both developing countries (Model 1) and developed countries (Model 2).

Model 1:

(1) $$y_{1}=x_{0}+b_{1}x_{1}+b_{2}x_{2}+b_{3}x_{3}+b_{4}x_{4}+b_{5}x_{5}+b_{6}x_{6}+\cdots +b_{9}x_{9}+u$$

Model 2:

(2) $$y_{2}=x_{0}+a_{1}x_{1}+a_{2}x_{2}+a_{3}x_{3}+a_{4}x_{4}+a_{5}x_{5}+a_{6}x_{6}+\cdots +a_{9}x_{9}+u$$

## 4. Methods and Data

### 4.1. Sample Selection

Based on the list of developed and developing countries in the press bulletin issued by the United Nations Conference on Trade and Development in 2005, 10 developing countries and 10 developed countries have been selected for analysis in this paper, as shown in Table 1. Data was chosen from 2004 to 2016 because, since the beginning of the 21st century, life expectancy has increased significantly and the world’s economy has reached an unprecedented level of development. Meanwhile, issues such as climate change and environmental pollution have become more prominent and now pose a huge threat to the health of mankind.

The classification of developed and developing countries was based on the results of the 2005 United Nations World Conference on Trade and Development, which classified Israel and South Korea as developed countries. The results have been widely accepted for some time. In this paper, 10 developing countries and 10 developed countries were selected for analysis, as shown in Table 1. These countries rely on the availability and validity of data; the countries are widely distributed in six continents with a large area, including five types of terrain and 11 types of climate that have typical environmental characteristics. In 2020, the total population of these countries was roughly 4.68 billion, accounting for about 60% of the world’s total population, which is representative to some extent. In the choice of developed countries, we chose countries with the world’s top GDP in the period 2004–2016, such as the United States, Japan, Germany, The United Kingdom, France, Italy, and Canada. These countries fully represent the economic scale of developed countries. In the choice of developing countries, we gave priority to countries with the highest GDP, such as China and India. Secondly, based on the comprehensive analysis of intercontinental distribution, the speed of economic development, trade types, and economic influence, we chose other countries such as Brazil, which has a free market economy and export-oriented economy, and Thailand, which has had a rapid economic development in recent years.

### 4.2. Data Sources

This paper uses Stata software to measure LE, and the Pearson analysis have been chosen to decompose the factors. Nine economic and environmental variables affecting the LE per capita have been selected, including five socio-economic variables and four environmental variables. The five socio-economic variables include GDP per capita, urbanization rate, current healthcare expenditure per capita, total public expenditure on education, and Gini coefficient. The four environmental variables include average annual exposure to PM_2.5_, CO_2_ emissions, fertilizer consumption, and forest area as a percentage of land area. The variables in this study have been chosen in order to focus on the level of economic development, ecology, and public policy, hence providing a socio-economic and ecological framework for increasing life expectancy.

Table 2 lists the data indicator meanings and database sources.

### 4.3. Design Methods

After comparing various research methods, this paper uses the Pearson Correlation Coefficient and multiple regression models to analyze influencing factors. The Pearson Correlation Coefficient provides a visual comparison of the degree of linear correlation between a factor under investigation and life expectancy and provides a basis for the development of regression models. Multiple regression models are widely applicable and commonly used in LE research to extract important information from a large amount of raw information and to mathematically model the relationship between variables so that the value of the dependent variable can be determined from the value of the independent variable. As LE is influenced by a number of factors, the multiple regression model is of great practical significance and is more suitable for exploring the specific relationship and the degree of influence between multiple factors and life expectancy.

This paper uses multiple regression models and the Pearson Correlation Coefficient not only to explore the relationship between multiple environment and economic variables on life expectancy and provide more support for future research, but also as a basis for making recommendations for countries to improve LE in order to achieve the sustainable development of human society.

LE per capita is a multi-factorial characteristic influenced by both socio-economic and environmental factors. Two models have been developed from the perspective of national development levels. Model 1 considers the mechanisms by which the economic development levels and environmental factors affect life expectancy in developing countries. Model 2 considers the mechanisms by which the economic development levels and environmental factors affect life expectancy in developed countries. The association and correlation between LE and the indicators of economic development levels and environmental factors in these models have been assessed using the Pearson Correlation Coefficient and multiple regression models.

In this study, LE per capita is influenced by nine selected variables, and a multiple linear regression model has been developed as follows [50]:

(3) $$y=x_{0}+c_{1}x_{1}+c_{2}x_{2}+c_{3}x_{3}+c_{4}x_{4}+c_{5}x_{5}+c_{6}x_{6}+\cdots +c_{9}x_{9}+u$$

Separate multiple linear regression models have been developed for developed countries and developing countries, where y represents LE at birth, *u* is a random disturbance term, and *x*_1_–*x*_9_ are all raw variables: *x*_1_, GDP per capita (in USD); *x*_2_, urbanization rate; *x*_3_, current healthcare expenditures per capita (in USD); *x*_4_, total public expenditures on education (total public expenditures on education as a percentage of GDP); *x*_5_, Gini coefficient; *x*_6_, average annual exposure to PM_2.5_ (micrograms per cubic meter); *x*_7_, CO_2_ emissions (metric ton per capita); *x*_8_, fertilizer consumption (kilograms per hectare of arable land); *x*_9_, forest area (forest area as a percentage of land area).

In addition, the two sets of scatter plots with nine variables have the correlation coefficients from the two models described above. From the scatter plots, it is possible to ascertain whether the correlation can be concluded.

## 5. Result

Table 3 presents the descriptive statistics and values of socio-economic and ecological indicators for both developing countries and developed countries. Our sample size is 130.

LE in developing countries ranged from 53.44 years in South Africa to 76.4 years in Thailand. The mean LE was 70.4 years, with a spread of 22.96 years. LE in developed countries ranged from 77.49 years in the United States to 83.98 years in Japan. The mean LE was 80.92 years, with a spread of 6.49 years.

GDP per capita in developing countries ranged from USD 2696 in India to USD 26,074 in Russia, with a mean of USD 12,454 and a spread of USD 23,378. GDP per capita in developed countries ranged from USD 23,785 in South Korea to USD 57,952 in the US, with a mean of USD 37,701 and a spread of USD 34,167.

Urbanization rate in developing countries ranged from 28.9% in India to 91.63% in Argentina. The mean LE was 60.74%, with a spread of 62.73%. Urbanization rates in developed countries ranged from 67.62% in Italy to 92.26% in Israel, with a mean of 81.46% and a spread of 24.64%.

Current health expenditures per capita in developing countries ranged from USD 25.06 in India to USD 1531 in Argentina, with a mean of USD 380.80 and a spread of USD 1505.94. Current health expenditures per capita in developed countries ranged from USD 717.90 in South Korea to USD 9878 in the United States, with a mean of USD 4041 and a spread of USD 9160.1.

Total public expenditures on education as a percentage of GDP in developing countries ranged from 2.029% in China to 6.314% in Brazil, with a mean of 4.006% and a spread of 4.285%. Total public expenditures on education as a percentage of GDP in developed countries ranged from 3.186% in Japan to 5.944% in Israel, with a mean of 4.876% and a spread of 2.758%.

The Gini coefficient for developing countries ranged from 32.70 in Indonesia to 64.80 in South Africa, with a mean of 44.95 and a spread of 32.1. The Gini coefficient for developed countries ranged from 17.40 in Germany to 42.50 in Israel, with a mean of 34.59 and a spread of 25.1.

Average annual PM_2.5_ exposure in developing countries ranged from 12.66 µg/m^3^ in Brazil to 99.81 µg/m^3^ in India, with a mean of 34.34 µg/m^3^ and a spread of 87.15 µg/m^3^. Average annual PM_2.5_ exposure in developed countries ranged from 6.549 µg/m^3^ in Canada to 34.32 µg/m^3^ in South Korea, with a mean of 15.79 µg/m^3^ and a spread of 27.771 µg/m^3^.

Carbon dioxide emissions in developing countries ranged from 0.770 t per capita in the Philippines to 12.62 t per capita in Russia, with a mean of 4.591 t per capita and a spread of 11.85 t per capita. Emissions in developed countries ranged from 4.573 t per capita in France to 19.66 t per capita in the United States, with a mean of 10.85 t per capita and a spread of 15.087 t per capita.

Fertilizer consumption in developing countries ranged from 11.42 kg/hm in Russia to 567.3 kg/hm in China, with a mean of 148.4 kg/hm and a spread of 555.88 kg/hm. Fertilizer consumption in developed countries ranged from 36.25 kg/hm in Australia to 643.4 kg/hm in Korea, with a mean of 199.5 kg/hm and a spread of 607.15 kg/hm.

Forest area as a percentage of land area in developing countries ranged from 7.618% in South Africa to 60.98% in Brazil, with a mean of 31.49% and a spread of 53.362%. Forest area as a percentage of land area in developed countries ranged from 7.116% in Israel to 68.48% in Japan, with a mean of 33.40% and a spread of 61.364%.

Table 4 and Table 5 present data from the economic development levels and environmental factors analysis for developed countries and developing countries, respectively. Based on the collective findings, it is safe to conclude that the influencing mechanisms from economic development levels and environmental factors on life expectancy may differ among different group of countries (see Table 4 and Table 5).

According to Table 4, in developing countries, significant positive correlations have been found between LE per capita and urbanization rate (Coefficient = 0.865, *p* = 0.001), total public expenditures on education as a percentage of GDP (Coefficient = 0.427, *p* = 0.001), and fertilizer consumption (Coefficient = 0.713, *p* = 0.001). At the same time, significant negative correlations have been found between LE per capita and GDP per capita (Coefficient = −0.340, *p* = 0.001), Gini coefficient (Coefficient = −0.912, *p* = 0.001), average annual exposure to PM_2.5_ (Coefficient = −0.467, *p* = 0.001), CO_2_ emissions (Coefficient = −0.323, *p* = 0.001), and forest area as a percentage of land area (Coefficient = −0.161, *p* = 0.008). No significant correlation has been found between LE per capita and current healthcare expenditures per capita (*p* = 0.187).

According to Table 5, in developed countries, significant positive correlations have been found between LE per capita and GDP per capita (Coefficient = 0.723, *p* = 0.001), urbanization rate (Coefficient = 0.629, *p* = 0.001), and forest area as a percentage of land area (Coefficient = 0.339, *p* = 0.001). Meanwhile, significant negative correlations have been found between LE per capita and current healthcare expenditures per capita (Coefficient = −0.923, *p* = 0.001), CO_2_ emissions (Coefficient = −0.825, *p* = 0.001), fertilizer consumption (Coefficient = −1.036, *p* = 0.001), and total public expenditures on education as a percentage of GDP (Coefficient = −0.174, *p* = 0.007). No significant correlation has been found between LE per capita and the Gini coefficient (*p* = 0.733) and the average annual exposure to PM_2.5_ (0.401).

**Figure 1:**
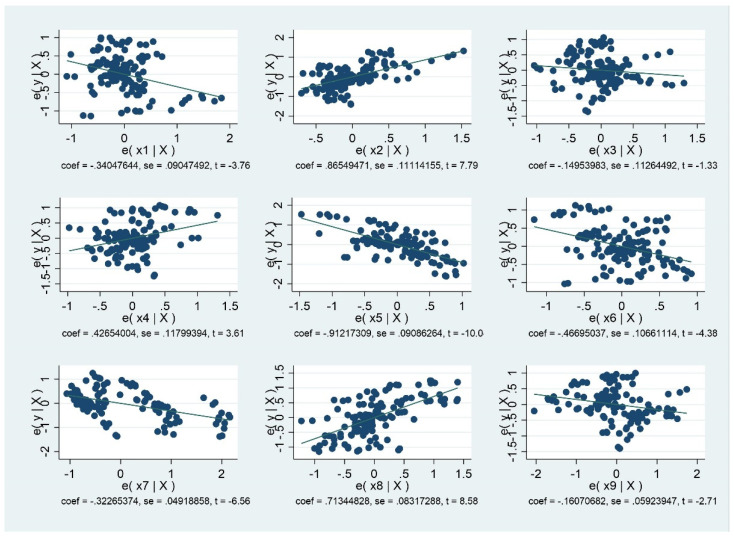
Scatter Plot of 9 Variables for Developing Countries.

**Figure 2:**
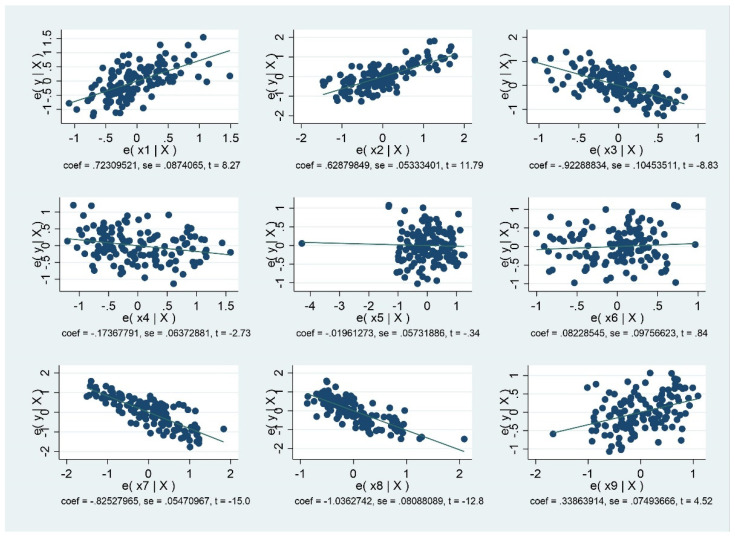
Scatter Plot of 9 Variables for Developed Countries.

From Figure 1 and Figure 2, it is possible to ascertain whether correlation coefficients are the correct tool to summarize the relationships. In addition, Figure 1 shows the correlation coefficients of 12 variables in developing countries. Figure 2 shows the correlation coefficients of 12 variables in developed countries.

Among developed countries, GDP per capita has the greatest positive impact on LE, while fertilizer consumption has the greatest negative impact on LE. Among developing countries, the urbanization rate has the greatest positive impact on LE, and the Gini coefficient has the greatest negative impact on LE.

Table 6 presents the multiple linear regression models for both developing countries (Model 1) and developed countries (Model 2).

Through our research, we have modeled the multiple linear regression models for both developing countries (Model 1) and developed countries (Model 2).

Model 1:

(4) $$y_{1}=-0.340x_{1}+0.865x_{2}-0.150x_{3}+0.427x_{4}-0.912x_{5}-0.467x_{6}-0.323x_{7}-0.713x_{8}-0.161x_{9}$$

Model 2:

(5) $$y_{2}=0.723x_{1}+0.629x_{2}-0.923x_{3}+0.174x_{4}-0.020x_{5}+0.082x_{6}-0.825x_{7}-0.104x_{8}+0.339x_{9}$$

## 6. Discussion

This study explores the differences in the impacts of the level of economic development and environmental factors on LE per capita in both developing countries and developed countries. The following conclusion can be safely drawn, based on the results from the above study.

GDP per capita has a significant impact in both developing countries and developed countries. In developed countries, high GDP per capita has a positive impact on life expectancy. In contrast, life expectancy at birth in developing countries is negatively correlated with GDP per capita, which is contradictory with most of the existing studies. Many existing research results show that there is a positive correlation between GDP per capita and life expectancy per capita. [6,21,22,43,46,59].

The urbanization rate has a positive impact on life expectancy in both developing countries and developed countries. The higher the urbanization rates, the higher the life expectancy. Thus, the urbanization rate can be seen as a tool for increasing life expectancy and improving living standards.

The impact of current healthcare expenditures per capita on life expectancy in developing countries and developed countries do not agree with the results of the majority of existing research. Many existing research results show that increased spending on healthcare can increase life expectancy of the population [6,16]. Increasing current healthcare expenditures per capita has a positive impact on life expectancy [60,61,62]. There is a negative impact on life expectancy in developed countries and there is no significant impact on life expectancy in developing countries. This reflects the fact that government expenditure on healthcare systems has not been as effective as expected. Therefore, a cost-benefit analysis should be done before implementing healthcare policies in order to achieve a better outcome. Cost-benefit analysis is conducive to the horizontal and vertical comparison of different periods and national health care policies, which can lead to reasonable cost control and resource allocation. However, it may also lead to inappropriate use of the cost-saving benefits of the incremental cost-benefit ratio. Cost-benefit analysis methods mainly include rationing and multi-standard system analysis. Countries should choose the appropriate method according to their health policy [63,64,65].

Total public expenditure on education as a percentage of GDP has a positive impact on life expectancy in developing countries. Thus, for developing countries, investment in education can be very effective in improving the population’s health conditions. However, our results show that the impact of total public expenditures on education as a percentage of GDP on life expectancy is not always positive. In developed countries, investment in education has a negative impact on life expectancy. As has been discussed previously, a cost-benefit analysis should be done for better outcome.

The Gini coefficient has a significant effect on life expectancy in developing countries, while it does not have the same effect on life expectancy in developed countries. These results support the threshold effect hypothesis, which assumes that there is a threshold of income inequality beyond which adverse effects begin to emerge. In developing countries with big income inequalities, income disparities have a negative impact on life expectancy. However, in developed countries, the Gini coefficient does not have a significant impact on life expectancy, mainly due to two reasons. Firstly, the income level in developed countries is relatively equal. Secondly, the welfare system in developed countries helps to mitigate the negative impact from the Gini coefficient on life expectancy. This indicates that the impact from income inequality on health conditions and life expectancy is not built in, and it may be affected by the different ways in which social and economic resources are allocated in developing countries and developed countries.

Average annual PM_2.5_ exposure has a significant effect on life expectancy in developing countries, but it does not have a significant effect on life expectancy in developed countries. One possible reason for this is that there is a threshold effect from annual mean PM_2.5_ exposure on life expectancy. In developing countries, the average annual PM_2.5_ exposure exceeds the threshold; hence, the average annual PM_2.5_ exposure contributes a negative impact on life expectancy. In developed countries, there is no significant effect on life expectancy. One possible reason for this is that the average annual PM_2.5_ exposure in developed countries is below the threshold. For example, in developing countries such as India, [66] the average annual PM_2.5_ exposure exceeds 35 μg/m^3^, 2–3 times that of the Temporary Target 1 of the World Health Organization [67], and most developed countries such as the United States have not exceeded the standard [68].

CO_2_ emissions have a significant negative impact on life expectancy in both developing countries and developed countries, indicating that the increase in greenhouse gas emissions will have a negative impact on life expectancy. From an environmental perspective, reducing CO_2_ emissions is crucial for increasing life expectancy globally.

The negative impact of fertilizer consumption on life expectancy in developed countries supports the point that soil pollution does have a negative impact on human beings’ health. However, the positive impact of fertilizer consumption on life expectancy in developing countries found in this study can also be explained by the positive correlation between fertilizer consumption and agricultural income. The increased agricultural income will, in return, positively affect life expectancy in developing countries. Additionally, in non-developed countries [69], reducing famine has a more positive effect on LE than healthy diet [70].

Forest area as a percentage of land area has a positive impact on life expectancy in developed countries, while the impact is negative in developing countries. In developed countries, the ability of the environment to self-purify, represented by the percentage of land area covered by forests, has a positive impact on the health conditions of the population. In developing countries, mainly because of the natural resources-curse phenomenon, the negative correlation between natural resources and government expenditures seriously affects the relationship between life expectancy and natural resources [71]. In short, the dependence on natural resources may negatively affect life expectancy in those countries with a higher than average value.

In conclusion, the results of our study show that, among developed countries, GDP per capita has the greatest positive impact on LE and fertilizer consumption has the greatest negative impact on LE. Among developing countries, the urbanization rate has the greatest positive impact on LE, while the Gini coefficient has the greatest negative impact on LE. In order to improve LE, it is highly recommended that countries should take improving GDP per capita and urbanization as their priorities, reducing the Gini coefficient, formulating appropriate healthcare and education policies, coordinating the relationship between economic development and environmental protection, paying more attention to environmental protection, reducing environmental pollution, and improving the self-purification capacity of the environment.

## 7. Conclusions

In developing countries, our hypothesis that significant positive correlations exist between LE per capita and urbanization rate and total public expenditures on education as a percentage of GDP has been proven. Significant negative correlations have been found between LE per capita, the Gini coefficient, average annual exposure to PM_2.5_, and CO_2_ emissions.

At the same time, our hypothesis that there are significant positive correlations between LE per capita and fertilizer consumption has been refuted. Additionally, significant negative correlations have been found between LE per capita and GDP per capita and forest area as a percentage of land area; no significant correlation has been found between LE per capita and current healthcare expenditures per capita.

Meanwhile, in developed countries, our hypothesis that significant positive correlations have been found between LE per capita and GDP per capita, urbanization rate, and forest area as a percentage of land area has been confirmed. Additionally, significant negative correlations have been found between LE per capita and CO_2_ emissions and fertilizer consumption.

At the same time, our hypothesis that significant positive correlations have been found between LE per capita and GDP per capita has been refuted. Additionally, significant negative correlations have been found between LE per capita and current healthcare expenditures per capita and total public expenditures on education as a percentage of GDP; no significant correlation has been found between LE per capita and the Gini coefficient and the average annual exposure to PM_2.5_.

We put forward five constructive recommendations for developing countries. First, a cost-benefit analysis should be done before implementing healthcare policies in order to achieve a better outcome. Second, increasing investment in education. Third, taking measures aimed at closing the gap between rich and poor. Fourth, reducing PM_2.5_ and CO_2_ emissions. Fifth, in underdeveloped countries, reducing famine has a greater positive impact on health and LE than a healthier diet.

We put forward four constructive recommendations for developed countries. First, developing the economy and increasing per capita GDP. Second, increasing urbanization rate. Third, a cost-benefit analysis in healthcare and educational investment should be done for better outcome. Forth, reducing fertilizer consumption.

## 8. Limitations and Outlook

In the future, there will be expansion in the sample size of our study. The study will also focus on a prolonged time frame Lastly, further decomposition of indicators will be carried out for an improved research outcome.

## Figures and Tables

**Table 1:** Classification of Sample Countries by Development Level.

Development Level	Sample Countries	Intercontinental Distribution
Developing Countries	China, India, Brazil, Russian Federation, Indonesia Thailand, Argentina, Mexico, Philippines, South Africa	1 in Europe 5 in Asia 1 in Africa 2 in South America 1 in North America
Developed Countries	United States, Japan, Germany, United Kingdom, France Italy, Canada, Republic of Korea, Australia, Israel	4 in Europe 3 in Asia 2 in North America 1 in Oceania

**Table 2:** Data Indicator Meanings and Database Sources.

Variable	Data Indicators	Meaning	Database Sources	References
*y*	LE at Birth	Human health conditions	The Global Observatory (GHO) database under the World Health Organization (WHO) (http://www.who.int/healthinfo/mortality_data/en) (accessed on 1 February 2021)	Reynolds, M.M. & Avendano, M. Social Policy Expenditures and LE in High-Income Countries [51].
*x*_1_	GDP per capita, PPP (constant 2017 USD)	The impact of economic development on human health	World Bank database (https://data.worldbank.org.cn/indicator?tab=all) (accessed on 1 February 2021)	Mellor, J.M. & Milyo, J. Reexamining the Evidence of an Ecological Association between Income Inequality and Health [52].
*x*_2_	Urbanization Rate	The relationship between population structure and health	World Bank database (https://data.worldbank.org.cn/indicator?tab=all) (accessed on 1 February 2021)	Rogers, R.G. & Wofford, S. LE in Less Developed Countries—Socioecenomic Development or Public Health [53].
*x*_3_	Current Healthcare Expenditures per capita (in USD)	Relationship between hygiene status and health	The Global Observatory (GHO) database under the World Health Organization (WHO) (http://www.who.int/healthinfo/mortality_data/en) (accessed on 3 February 2021)	Zare, H., Gaskin, D.J. & Anderson, G. Variations in LE in Organization for Economic Co-operation and Development countries—1985–2010 [54].
*x*_4_	Total Public Expenditures on Education (Total Public Expenditures on Education as a Percentage of GDP)	The relationship between educational inputs and health	World Bank database (https://data.worldbank.org.cn/indicator?tab=all) (accessed on 4 February 2021)	Meara, Ellen R; Richards, Seth; Cutler, David M. The Gap Gets Bigger: Changes in Mortality and LE by Education, 1981−2000 [55]. Reynolds, M.M. & Avendano, M. Social Policy Expenditures and LE in High-Income Countries [51].
*x*_5_	Gini Coefficient	The relationship between income inequality and health	World Bank database (https://data.worldbank.org.cn/indicator?tab=all) (accessed on 1 February 2021)	Kim, J.I. & Kim, G. Effects on inequality in LE from a social ecology perspective [50]. Ross, N.A. et al. Relation between income inequality and mortality in Canada and in the United States: cross sectional assessment using census data and vital statistics [56].
*x*_6_	Average Annual Exposure to PM_2.5_ (micrograms per cubic meter)	The impact of air pollution on LE	World Bank database (https://data.worldbank.org.cn/indicator?tab=all) (accessed on 2 February 2021)	Wen, M. & Gu, D. Air pollution shortens LE and health expectancy for older adults: The case of China [57].
*x*_7_	Carbon Dioxide Emissions (metric tons per capita)	Impact of greenhouse gas emissions on LE	World Bank database (https://data.worldbank.org.cn/indicator?tab=all) (accessed on 1 February 2021)	Cheng, Q.; Li, M.; Li, F.; Tang, H. Response of Global Air Pollutant Emissions to Climate Change and Its Potential Effects on Human LE Loss [49].
*x*_8_	Fertilizer Consumption (kg per hectare of arable land)	The impact of soil contamination on LE	World Bank database (https://data.worldbank.org.cn/indicator?tab=all) (accessed on 4 February 2021)	Sharma, N. & Singhvi, R. Effects of Chemical Fertilizers and Pesticides on Human Health and Environment: A Review. *International Journal of Agriculture* [58].
*x*_9_	Forest Area (forest area as a percentage of land area)	The role of environmental self-purification capacity in health	World Bank database (https://data.worldbank.org.cn/indicator?tab=all) (accessed on 3 February 2021)	Zha, X., Tian, Y., Gao, X., Wang, W. & Yu, C. Quantitatively evaluate the environmental impact factors of the LE in Tibet, China [9].

**Table 3:** Descriptive Statistics of Variable (All values are from the period 2004–2016). Top: Developing countries. Bottom: Developed countries. Mean: The average value. StDev ^a^: Based on sample estimation standard deviation, which reflects the degree of discrete relative to the average value (mean). Minimum: The data in the same group to the minimum values. Maximum: The data in the same group to the maximum values.

Variable	Mean	StDev ^a^	Minimum	Maximum
*y*	70.47	5.376	53.44	76.40
*x*_1_	12,454	5320	2696	26,074
*x*_2_	60.74	19.20	28.90	91.63
*x*_3_	380.8	339.4	25.06	1531
*x*_4_	4.006	1.199	2.029	6.314
*x*_5_	44.95	8.325	32.70	64.80
*x*_6_	34.34	25.21	12.66	99.81
*x*_7_	4.591	3.411	0.770	12.62
*x*_8_	148.4	131.7	11.42	567.3
*x*_9_	31.49	16.79	7.618	60.98
*y*	80.92	1.473	77.49	83.98
*x*_1_	37,701	6844	23,785	57,952
*x*_2_	81.46	6.307	67.62	92.26
*x*_3_	4041	1802	717.9	9878
*x*_4_	4.876	0.689	3.186	5.944
*x*_5_	34.59	3.681	17.40	42.50
*x*_6_	15.79	6.615	6.549	34.32
*x*_7_	10.85	4.319	4.573	19.66
*x*_8_	199.5	113.8	36.25	643.4
*x*_9_	33.40	19.08	7.116	68.48

**Table 4:** Descriptive Statistics of Variable (Up: Developing Countries; Down: Developed Countries).

Variables	Coefficient	t-Value	*p*-Value	R-Squared
*x*_1_	−0.340	−3.76	0.000	0.7617
*x*_2_	0.865	7.79	0.000	
*x*_3_	−0.150	−1.33	0.187	
*x*_4_	0.427	3.36	0.000	
*x*_5_	−0.912	−10.04	0.000	
*x*_6_	−0.467	−4.38	0.000	
*x*_7_	−0.323	−6.56	0.000	
*x*_8_	0.713	8.58	0.000	
*x*_9_	−0.161	−2.71	0.008	

**Table 5:** Multiple Linear Regression Coefficients for Developed Countries.

Variables	Coefficient	t-Value	*p*-Value	R-Squared
*x*_1_	0.723	8.27	0.000	0.8054
*x*_2_	0.629	11.79	0.000	
*x*_3_	−0.923	−8.83	0.000	
*x*_4_	−0.174	−2.73	0.007	
*x*_5_	−0.020	−0.34	0.733	
*x*_6_	0.082	0.84	0.401	
*x*_7_	−0.825	−15.08	0.000	
*x*_8_	−1.036	−12.81	0.000	
*x*_9_	0.339	4.52	0.000	

**Table 6:** Multiple Linear Regression Models for both Developing Countries and Developed Countries.

Model 1	
*y*_1_ = −0.340*x*_1_ + 0.865*x*_2_ − 0.150*x*_3_ + 0.427*x*_4_ − 0.912*x*_5_ − 0.467*x*_6_ − 0.323*x*_7_ − 0.713*x*_8_ − 0.161*x*_9_	*R*^2^ = 0.7617, *F*-Value = 42.62, *p* = 0.001
**Model 2**	
*y*_2_ = 0.723*x*_1_ + 0.629*x*_2_ − 0.923*x*_3_ − 0.174*x*_4_ − 0.020*x*_5_ + 0.082*x*_6_ − 0.825*x*_7_ − 0.104*x*_8_ + 0.339*x*_9_	*R*^2^ = 0.8054, *F*-Value = 55.19, *p* = 0.001

## Data Availability

Publicly available datasets were analyzed in this study. This data can be found here: The Global Observatory (GHO) database under the World Health Organization (WHO) (http://www.who.int/healthinfo/mortality_data/en) (accessed on 1 February 2021), World Bank database (https://data.worldbank.org.cn/indicator?tab=all) (accessed on 1 February 2021).
